# Models for the retention of duplicate genes and their biological underpinnings

**DOI:** 10.12688/f1000research.141786.1

**Published:** 2023-10-24

**Authors:** Raquel Assis, Gavin Conant, Barbara Holland, David A. Liberles, Malgorzata M. O'Reilly, Amanda E. Wilson

**Affiliations:** 1 Florida Atlantic University, Boca Raton, Florida, USA; 2 North Carolina State University, Raleigh, North Carolina, USA; 3 University of Tasmania, Hobart, Tasmania, Australia; 4 Temple University, Philadelphia, Pennsylvania, USA

**Keywords:** gene duplication, probabilistic modeling, theoretical biology, Markov model, synteny, phylogenetic analysis

## Abstract

Gene content in genomes changes through several different processes, with gene duplication being an important contributor to such changes. Gene duplication occurs over a range of scales from individual genes to whole genomes, and the dynamics of this process can be context dependent. Still, there are rules by which genes are retained or lost from genomes after duplication, and probabilistic modeling has enabled characterization of these rules, including their context-dependence. Here, we describe the biology and corresponding mathematical models that are used to understand duplicate gene retention and its contribution to the set of biochemical functions encoded in a genome.

## Introduction

Richard Feynman left the quotation, “What I cannot create, I do not understand” on his blackboard at the time of his death. Creation in mathematical modeling is writing down a model that describes a process. The retention of duplicate genes over long evolutionary periods involves mechanisms and processes in population genetics, evolution, molecular biology, ecology and biochemistry. Here we describe the current state of modeling in the field of duplicate gene retention.

The genome can experience duplications of its content across a range of sizes, from incomplete duplications of single genes to small-scale events (single gene duplicates through tandem duplication or retrotransposition events) to larger scale events that involve multiple genes or even whole genomes. These events are broadly classified as whole genome duplications (WGD) or small scale duplications (SSD): the two types have several key differences. WGD duplicates are duplicated together with their interacting partners, and the population (and cellular) genetic model for the initial fixation of WGD duplicates is less straightforward than for SSDs. WGD events can be either allopolyploidies or autopolyploidies (see below). SSD duplicates typically do not see their interaction partners duplicated and initially have a frequency of 1/2N in a diploid population. SSD duplicates are typically assumed to begin as identical copies, but this isn’t always the case. ^1^^–^^6^

Once duplicated, the accumulation of nonfunctionalizing mutations leading to the eventual loss of one copy through neutral processes can be naturally modeled with an exponential decay distribution. ^7^^–^^10^ Several factors can lead to duplicate pairs losing their redundancy and falling under the action of natural selection; when this change occurs that pair will deviate from the neutral expectation of exponential loss. ^10^^–^^12^ In general, it has been suggested by Wagner (2005) ^13^ that the expression cost of a duplicated gene leads to a fitness cost to its possessor, and a similar argument could be made for a replication cost, especially for organisms that have limitations on genome size. Such a limitation would occur when selection acts to minimize replication time in log phase growth.

More generally, duplicates can be retained as a mechanism to gain extra expression. ^14^ An example of this in humans and other mammals appears to be the convergent duplication and retention of copies of the amylase gene. ^15^ Trypanosomes seem to regulate gene expression more generally through gene duplication, with very few transcription factors in their genomes. ^16^^,^^17^ There is also a selective pressure to retain duplicates that physically interact in stoichiometric balance to prevent misassembly of imbalanced heteromultimers or pathological interactions among the exposed hydrophobic surfaces of those multimers. ^18^^–^^22^ Mechanisms of duplicate preservation that involve changes of function through mutation accumulation include subfunctionalization, ^23^^,^^24^ the partitioning of functions among copies from the pre-duplication ancestral state, and neofunctionalization, ^14^ the acquisition of a new beneficial function. Multiple models for all of these processes exist and are described below together with their biological assumptions.

## Gene Duplicability

These different mechanisms of retention are differently applicable to different genes. This has given rise to the notion of gene duplicability, that some genes are inherently more duplicable than other genes. ^8^^,^^25^^,^^26^ For a gene to be retained after duplication, it ultimately needs to be either subfunctionalizable or neofunctionalizable. ^10^ To be subfunctionalizable, a gene must have at least two modular functions (biochemical activities, including binding, or modular expression domains) such that there are mutations that can disable one subfunction without damaging others. The probability of eventual subfunctionalization for two identical duplicate copies scales as 1-(0.5)^(f-1), where f is the number of functions. The probability of neofunctionalization is harder to quantify, but either new selectable biochemical functions or expression domains must be evolvable. At the coding sequence function level, this is influenced by the fold, the number of binding partners, including those that are obligate heteromultimerization partners, ^20^^,^^21^ and the type of function encoded. ^27^^–^^33^ The network position and expression level also influence gene duplicability. ^26^^,^^31^^,^^34^^–^^38^ Empirically, there is a class of “duplication-resistant” genes where natural selection apparently acts against the maintenance of both copies. ^33^ Different genomes might have different proportions of genes that are duplicable as well as that are subject to dosage constraints. ^39^

In analyzing the retention of genes following two rounds of whole genome duplication, the Atlantic salmon genome paper ^40^ presented a conditional probability analysis suggesting that the gene duplicability hypothesis predicts that genes retained after one round of genome duplication might be more likely to be retained after the second round. However, prior analyses from plants tended to suggest that the factors that favor the retention of duplicates after a first polyploidy tend to be attenuated in subsequent polyploidies. ^41^ Support for the hypothesis from the test in Atlantic salmon was also lacking, but there is more complexity to the process, including changing gene duplicability, the time-dependence of the retention process, and other factors ^42^^,^^43^ and probabilistic models that can be used as an expectation for different hypotheses are described below. ^39^

## The Biological Considerations as Building Blocks for Models

To model duplicate gene retention, one must describe what is mutable and selectable. Protein encoding genes must function as proteins after transcription and translation. They are expressed at a given concentration in specific places and at specific times. They then carry out various functions: binding, catalysis, or transport in interaction with other molecules in the cell. This is what we mean by function. The expression domains for a protein are a quantitative description of where and when expression occurs. There are cases where duplication is used as a mechanism of amplifying expression level, although this seems to be a temporary situation in most organisms, with trypanosomes being a possible exception.

## Modeling Expression Evolution of Duplicate Genes as a Stand-Alone Process

Though classical models for the retention of duplicate genes often consider their levels of sequence divergence, gene expression data provide a promising source of underutilized information. In particular, gene expression data are now widely available for many species and often consist of measurements across multiple conditions, which can include tissues, sexes, and developmental stages. These measurements are an attribute of function, as knowledge of where and when a gene is expressed provides insight into its biological roles. Indeed, Ohno proposed that the first step of functional divergence between duplicate genes is their expression divergence. ^14^ Thus, gene expression is a trait that can be exploited to understand gene function and, in the case of duplicate genes, the divergence between their functions.

Many early studies compared expression levels between duplicate genes, finding that divergence between copies is often widespread, rapid, and asymmetric. ^11^^,^^44^^–^^46^ Yet expression divergence between duplicate genes does not provide information about the exact mechanisms of their retention. For instance, both neofunctionalization and subfunctionalization result in functional divergence between gene copies. Thus, it is important to compare the expression profiles of both copies to that of the ancestral single-copy gene, as this can elucidate how each copy has changed since duplication. Such an approach was developed about a decade ago ^47^ and later implemented as the software CDROM. ^48^ Applications of this approach have uncovered widespread neofunctionalization in *Drosophila,* ^47^ mammals, ^49^ honeybees, ^50^ and grasses. ^51^

However, a key shortcoming of the approach of Assis and Bachtrog (2013) ^47^ is that it does not account for stochastic changes in gene expression arising from phenotypic drift. ^52^ This obstacle can be overcome by modeling gene expression evolution on the phylogenetic tree relating a pair of duplicates and their single-copy ancestor. There is a natural hierarchy of models for describing how gene expression evolves along the branches of a phylogenetic tree. The simplest is Brownian motion (BM), which models phenotypic drift without making any assumption of selection for a particular expression level. ^53^ The next level of the hierarchy is an Ornstein-Uhlenbeck (OU) process with stabilizing selection for a particular expression level. ^54^^–^^58^ Finally, one can utilize an OU process with a shift reflecting positive selection for an optimal expression level. ^59^^–^^63^ Another conceivable approach is to model the genotype driving expression evolution, but this requires an understanding of the combinatorial role of promoters and enhancers regulating expression. ^64^^,^^65^ which is currently beyond our modeling capabilities. ^66^

With this in mind, researchers have recently begun to employ OU models for studying the expression evolution of duplicate genes. ^22^^,^^67^ Additionally, DeGiorgio and Assis (2021) ^67^ developed CLOUD, which also predicts retention mechanisms of duplicate genes by overlaying their OU model with a neural network. ^67^ Though likelihood ratio tests (LRTs) have classically been used for similar tasks with single-copy genes, ^22^^,^^56^^–^^59^^,^^68^ machine learning approaches present several advantages, such as the optimization of model fit to training data, direct evaluation of performance on independent test data, and ability to make predictions from data with correlated or conflicting signals. ^69^ Further, such methods make predictions solely from data, ^69^ which can be advantageous when the underlying evolutionary model is unknown. Indeed, CLOUD demonstrates excellent predictive performance, outshining CDROM in classifying retention mechanisms while also being able to predict parameters corresponding to expression optima and strengths of selection and drift. ^67^

Still, much remains to be done in this area. For one, many advanced machine learning algorithms have yet to be explored in this context. Moreover, though expression data can provide a lot of useful information about a gene, this does not necessarily mean that we should neglect other complementary sources, such as its sequence or protein structure. An additional advantage of machine learning is that it is not weighed down by additional information, as correlated or conflicting signals can be reduced or even removed through regularization. However, the problem lies in extending the underlying OU model to accommodate diverse pieces of information. Last, one can argue that the most important extension of such work is to accommodate more species and gene copies. Currently, most researchers do not have access to expression data for multiple of the same conditions in many species, but this is soon to change as the cost of sequencing continues to decrease. And of course, it is critical to assay the fates of gene families with more than two members, as many such families are prominent across study systems and may be key to understanding adaptation.

## Modeling the Evolutionary Cost of Gene Duplication

Analyses of duplicated genes often start from the premise that gene duplications are selectively neutral, creating redundant copies that can potentially degrade through degenerative mutations. ^7^^,^^70^ The patterns of which genes do and do not tend to survive in duplicate and the dosage-balance hypothesis (see above) already suggest that gene duplications are not all necessarily selectively neutral at birth. As mentioned above, Wagner has extended this argument by showing that, at least in microbial organisms, the gene expression costs associated with an extra gene copy are rarely if ever expected to be selectively neutral. ^13^^,^^71^ His model considers the per-time unit cost, in terms of high energy phosphate bonds, of expressing a duplicated gene for different ranges of mRNA and protein levels, finding that, for reasonably large values of the effective population size of microbial species, those energy costs are large enough to discount the hypothesis that the duplication’s fate is primarily driven by neutral evolution. ^72^ The principle that excess gene expression has measurable negative fitness effects due to the costs of transcription and translation has been elegantly experimentally explored in *E. coli.* ^73^

## Dosage Imbalance Cost

It is well established that maintaining stoichiometric balance with interacting partners is an important driving force to preserve duplicate genes in genomes while waiting for other preservation mechanisms to act. The mechanistic driving force behind this is thought to be the prevention of the accumulation of exposed hydrophobic residues that populate binding interfaces and can lead to misinteractions that might be deleterious to cells. An explicit model that relates fitness to the expected concentration of surface hydrophobic residues has been generated and used to explore how this model enables the transition to subfunctionalized states, with opposite trends observed after whole genome duplication and smaller scale duplication. ^74^ This model is a mechanistic update over a previously described hazard function model that didn’t model this underlying biochemistry. ^75^

## Population Genetic Considerations

In Eukaryotic organisms, the baseline state for most chromosomes is diploidy. Either across the whole genome or for individual loci, this diploid state is disrupted after gene duplication. Functional tetraploidy has meiotic implications that are not present for smaller scale duplication events (see ^40^ for a discussion in Atlantic salmon). Over time, whole genome duplication events return to a state of functional diploidy and may start that way for alloduplication events with the chromosome sets already diverged, as may have been the case for Xenopus ^76^ and Brassica species. ^77^^,^^78^

However, so far, this divergence characterization has been viewed without the underlying population-level dynamics. While more complex for whole genome duplication events, smaller scale duplicates in diploid organisms begin with a frequency of 1/2N and must fix before they are lost if they are going to be retained. The neutral expectation for eventual fixation for such duplicates is that they will fix with a probability equal to their frequency. Classical population genetics then gives a time-dependent expectation for the frequency based upon the age of the duplicate. Stark *et al*. (2021) ^79^ have presented a population genetic model to evaluate if the age-dependent frequency is unexpected for a duplicate evolving neutrally. The power of the approach was evaluated using a Moran Model, with two selective parameters, one for selection on the duplicate itself, which can be positive when total gene dosage amplification is beneficial or negative due to factors like expression and replication cost, and the other for selection on changed function (for example neofunctionalization).

In using this model with actual segregating duplicates, the frequency of a duplicate in a population can be measured through population genomic sequencing. However, the age of the duplicate is the next question. For SNPs, population geneticists examine the length of tracts of identity by descent to estimate the age of an allele (e.g. duplicate locus) (see ^80^), but a much simpler approach based upon pairwise pS values between copies may be possible. Application of these approaches to real data hasn’t been performed yet to evaluate their performance.

Those considerations are used to evaluate selection on the copy itself. Selection on the sequence of the duplicate might be measured by a more rich data source and parametrization or in a simpler manner by examining ratios like pN/pS or in using tests like the McDonald-Kreitman Test applied to duplicates. ^81^

## Interspecific and Phylogenetic Models

Moving from intraspecific to interspecific analysis of gene duplicates, including models that run on a single genome, early work from Lynch and Conery (2000) ^7^ and from Lynch, Force, and coworkers ^8^^,^^23^^,^^82^ has been pioneering. The first model presented by Lynch and Conery (2000) ^7^ modeled duplicate gene retention expectations with a simple exponential distribution. This assumes that no matter how long a gene has been in a genome, the instantaneous probability of loss is constant, which is not consistent with retention mechanisms, but is useful as a non-functionalization null model. Konrad *et al*. (2011) ^10^ and Teufel *et al*. (2016) ^75^ described sets of hazard functions that did not have the property of time-independent hazard functions and Zhao *et al*. (2015) ^83^ presented an age-dependent birth-death process inspired by this framework. Yohe *et al*. (2015) ^84^ presented a theoretical gene tree-species tree reconciliation framework using the Konrad *et al*. (2011) ^10^ model, but this was never implemented as software. Arvestad *et al*. (2009) ^9^ presented a formal probabilistic gene tree-species tree reconciliation framework using the exponential distribution model and generated software for this. Others have created similar software packages. ^85^^,^^86^ Additional innovations to this framework have included the treatment of synteny, ^87^ and species level processes. ^88^

Contemporaneously with the Lynch and Conery (2000) ^7^ modeling, Force *et al*. (1999) ^23^ presented a more mechanistic framework for subfunctionalization and neofunctionalization as processes. This was formalized as a Markov Model by Stark *et al*. (2017) ^89^ and expanded upon by Wilson and Liberles (2023) ^74^ to enable consideration of dosage balance. Multi-scale Markov models reflect a further step in this trajectory.

## Multi-Scale Markov Models

A wide range of multi-scale Markov models for the evolution of gene families have been studied in the literature. Models with states that record very detailed information about biology are suitable for simulation-based analysis. However, such models may not be useful for theoretical analysis, due to the size of their state space. On the other hand, models with simplified state space are useful for in-depth theoretical and numerical analysis which often leads to novel biological insights. Both types of models provide powerful tools for the analysis, and the choice of one over another may depend on the type of biological questions one might want to answer.

As an example, Stark (2017) ^90^ suggested a simulation model for the evolution of the family of genes, in which detailed information is recorded within a binary-matrix of 0’s and 1’s such that each row corresponds to a gene and each column corresponds to its function. Later, Diao *et al*. (2022) ^91^ applied the binary-matrix model of Stark (2017) ^90^ in their simulation-based analysis which led to some interesting biological insights. Their results suggested that when the rate of gene duplication dominates the rate of gene loss, then the distribution of tree shapes is close to following the uniform ranked tree shape (URT) distribution (i.e. the distribution for a constant birth-death process). However, when the opposite is true then gene trees are more balanced than the URT distribution.

**Figure 1.**
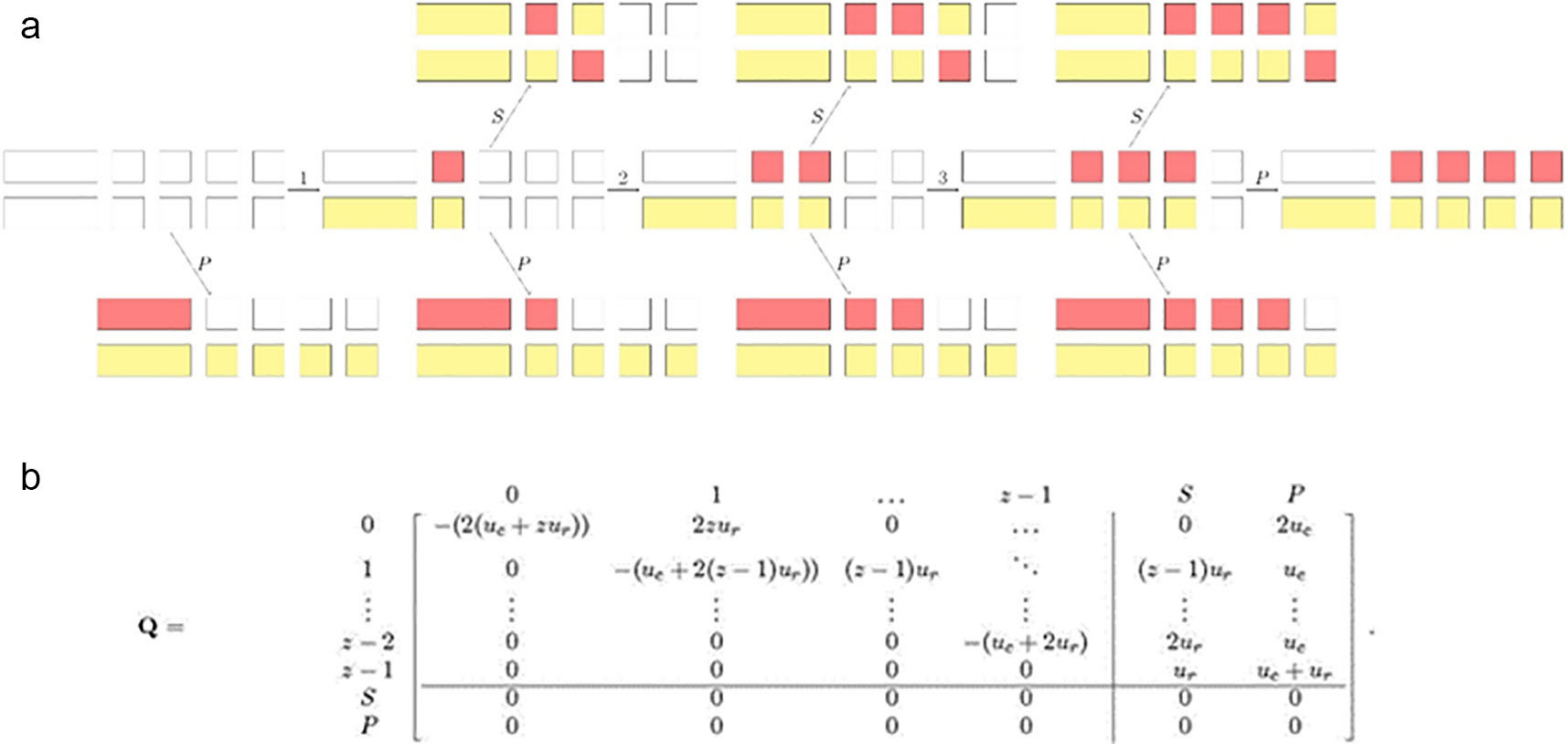
To model the evolution of gene duplicates, Stark *et al*. (2017) ^89^ constructed a Markov chain with state space {0,1, …,z-1,S,P} and generator **Q** where z is the number of regulatory regions within the gene, and S and P is are the subfunctionalization and the pseudogenization (absorbing) state, respectively. In the above example of transitions with z=4, the regions hit by null mutations are in red, and the regions protected by selective pressure are in yellow. This figure is adapted from Stark *et al*. (2017), ^89^ which was published under an open access license.

Next, Stark *et al*. (2017) ^89^ constructed a model with a simplified state space, for the theoretical analysis of the evolution of gene duplicates, see Figure 1. The manageable size of the state space allowed them to derive analytical expressions for the rates of subfunctionalization and pseudogenization. This led to the result, earlier predicted by classical models, that models with subfunctionalization provided a better fit to the age distribution of duplicate genes than models with a constant rate. Further, Diao *et al*. (2020) ^92^ developed a more advanced model for the theoretical analysis of the evolution of a family of gene duplicates, based on the application of a level-dependent Quasi-Birth-and-Death process (QBD). The state (n,m,k) of their QBD model consists of the variables n and m representing the number of genes and the number of the redundant genes respectively, and the variable k which, in a simplified manner, records the remaining information about the family. The authors took advantage of both types of models. They used the simulation-based binary-matrix model of Stark (2017) ^90^ to obtain detailed outputs and then fitted the parameters of their QBD to data obtained from these outputs. Next, they derived biological insights by computing metrics based on the expressions from the theory of QBDs, such as the stationary distribution of observing various states within the model and the distribution of the time it takes for the family to lose a gene. Soewongsono *et al*. (2023) ^93^ then applied this QBD model to a more general problem of reconciliation, in which the task is to find a mapping of a gene tree into a species tree, to maximize the likelihood. The authors provided an algorithm to compute the likelihood of the reconciliation, given the available incomplete data.

## Modeling Asymmetric Divergence and Gene Conversion in Duplicated Genes

Another angle to evaluate duplicate gene retention mechanisms involves an examination of evolutionary symmetry. In fact asymmetric divergence between pairs is relatively common. ^94^ This result is particularly striking when one considers that many tests have low power to detect asymmetry. ^95^ The test that was employed uses an outgroup gene from a relative lacking the duplication to polarize the differences between the two paralogs, forming a triplet tree. Maximum likelihood was then used to compare a symmetric model, where dS (K), dN (K _a_), or dN/dS (also K _a_/K _s_ or omega) was constrained to be equal for both paralogs to an alternative model where the divergence statistic is allowed to differ between paralogs. ^94^ It is tempting to attribute to neofunctionalization cases where one paralog has accelerated evolution relative to the other, but in fact many different modes of evolution can induce asymmetry. ^96^ In fact, asymmetry in divergence is arguably more interesting for the light it sheds on other questions regarding duplicate evolution, such as the differences seen between duplicated genes created by different mechanisms ^3^ or as a means for detecting recent gene conversion. We have used the same triplet-based approach to show that duplicated ribosomal proteins created by the ancient polyploidy in bakers’ yeast have experienced very recent gene conversion events, such that ancient polyploidy-produced paralogs in one species are more similar to each other than those copies are to their orthologs in a closely related species, despite the fact that the divergence time between the ortholog pairs is probably ten-fold less than the time since the paralogs were formed. ^97^

Gene conversion refers to several mutational mechanisms that can allow part of the sequence of one member of a gene family to overwrite the corresponding region in another paralog, effectively erasing some of the divergence between those two paralogs. ^98^ Because such events violate the assumption of independent evolution between paralogs, they are difficult to treat with standard models. Ji *et al*. (2016), ^99^ have described a codon model of evolution that jointly considers the paired codons from two paralogous genes, incorporating a parameter t modeling the frequency with which conversion events alter the paralogs’ sequences. This model confirms the surprisingly high rate of gene conversion among the yeast ribosomal proteins, which had previously and incorrectly been taken to represent the more general rate of gene conversion among yeast paralogs. ^100^

## Whole Genome Duplication: Duplicate Losses, Modeling and Synteny

While approaches such as standard time-independent birth and death models can be applied to duplicate genes produced by whole-genome duplications (WGD), or polyploidies, there are complexities and opportunities introduced by WGD events that benefit from models that are specific to them. Polyploidy refers to a variety of events that result in eukaryotic cells with more than two copies of the genome. ^101^ Polyploid lineages are formed relatively often but most quickly go extinct. ^102^ However, great trunks of the eukaryotic tree of life descend from surviving ancient polyploidy events, including all vertebrates and flowering plants, as well as lineages of yeasts, ciliates and other plants. ^103^

Polyploid individuals can form through the merger of genomes from the same species, known as autopolyploidy, or of distinct species, referred to as allopolyploidy. ^104^ The relative frequency of formation of these two types of polyploids many be approximately equal, ^105^ but because allopolyploidy confers the potential benefits of both polyploidy and hybridization, there is reason to suspect that most surviving ancient polyploidy events were allopolyploidies. ^105^^–^^107^

The phrase whole-genome duplication is potentially slightly misleading because it suggests that all genes in the genome are duplicated. Initially they are. However, for any reasonably old polyploidy event, many or even most of the duplicated genes will have been lost. ^107^^,^^108^ Probably most of these losses occur through the fixation of loss-of-function mutations in one copy by genetic drift, a process common to duplicates of all types. ^7^ As described, selection from various sources can also play a role.

## Another Class of Models for Evolution After Polyploidization Events

The question of demonstrating that a particular genome has an ancient polyploidy in its history is a complex one ^109^ and somewhat distinct from our concerns here. However, one obvious consequence of a polyploidy is the production of a group of duplicated genes that were all formed “at the same moment.” In principle, a neutral measure of paralog divergence, such as the number of synonymous substitutions per synonymous site (K _s_) should be able to detect a polyploidy through the excess of duplicates with similar K _S_ values. ^7^^,^^45^ While the actual practice of detecting polyploidy events in this way is one that requires care, ^110^ it has been an extremely illuminating approach. For instance, in a pioneering study, Maere *et al*. (2005), ^111^ were able to fit a mixture of age models to the *Arabidopsis thaliana* genome and detect three different ancient polyploidies in its history. They further showed convergent retention of genes of similar function in duplicate after these different events. ^111^ To do so, they modeled three distinct processes: 1) a basal continuous rate of single gene duplication, 2) a set of between one and three ancient polyploidy events and 3) continuous losses of duplicates created by 1 and 2. They evolved these three processes in simulated discrete K _s_ time intervals and fit the simulations to the observed set of K _s_ values from duplicated genes found in the *A. thaliana* genome.

Maere *et al*.’s (2005) ^111^ approach is elegant but challenging to implement: other analyses of a similar form have instead fit mixtures of models to the observed duplicate divergences, combining a basal steady-state duplicate birth-death model with one or more discrete events duplicating the entire genome (with the potential for the immediate removal of some of these duplicates). ^110^^,^^112^ Such approaches allow for testing hypotheses regarding the number of polyploidy events in the lineage of a genome, but the results require some caution in their interpretation due to the relatively modest information provided by K _s_ values. ^110^

## Polyploidy and Gene Synteny

**Figure 2.**
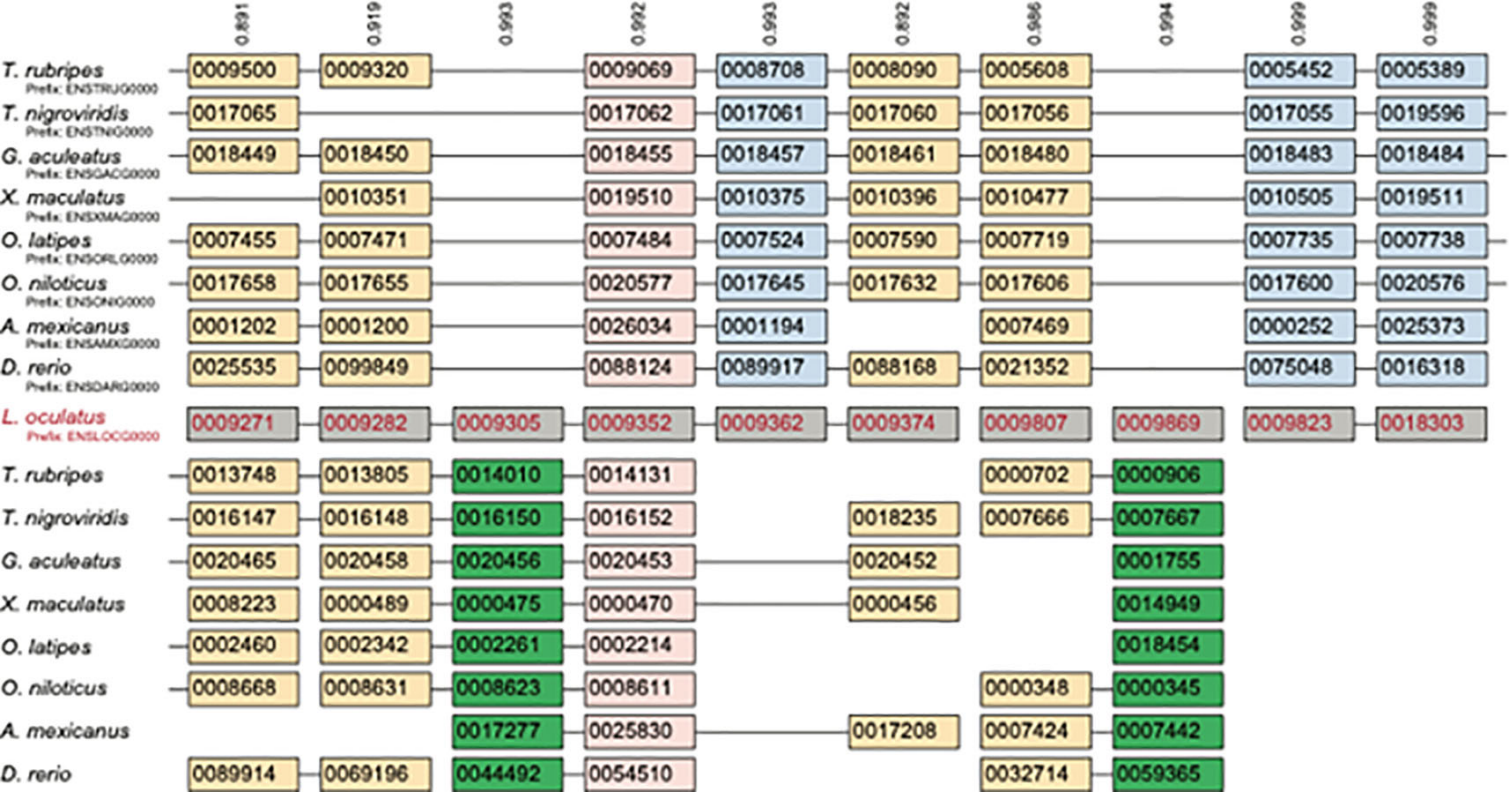
A region of ten ancestral genes duplicated through the teleost-specific genome duplication (TGD). ^123^ Shown in the center in gray are the ten genes as they are found in the genome of the spotted gar ( *L. oculatus*), which lacks the TGD. The lines joining pairs of genes indicate that these genes are neighbors in the genome (i.e., they are in synteny). After the TGD, some duplicates survive in all (pink) or some (tan) genomes, while others have been returned to single copy, either from the subgenome with more surviving genes (blue) or than with fewer (green). Numbers at the top of each column/pillar are the orthology confidence estimates from POInT. Genes are shown with their Ensemble identifiers ^124^ for reference. This figure is an original figure produced by the authors for this review article.

Our discussion so far has considered signals such as gene tree topologies and divergence measures which are applicable to all types of duplicated genes. However, for the specific case of a polyploidy, another type of highly informative data are present: the gene order patterns among the duplicated and non-duplicated genes. These patterns are commonly referred to as gene *synteny.* They were critical in identifying the first ancient polyploidy found in a eukaryotic genome ^113^ and have been used in many subsequent analyses of polyploid genomes. ^114^^–^^116^ Figure 2 illustrates the principle that a genome duplication, in contrast to single-gene duplications, produces duplicated genes that preserve the gene order present in the unduplicated ancestor. Indeed, these patterns can identify ancient polyploidies even in the limiting case where all of the duplicated genes were subsequently lost, provided an outgroup genome lacking the polyploidy is available and the degree of chromosomal rearrangement is not too large. Hence, synteny is often considered the best evidence of the presence of an ancient polyploidy, even if formal tests using it are hard to develop. ^110^^,^^117^

**Figure 3.**
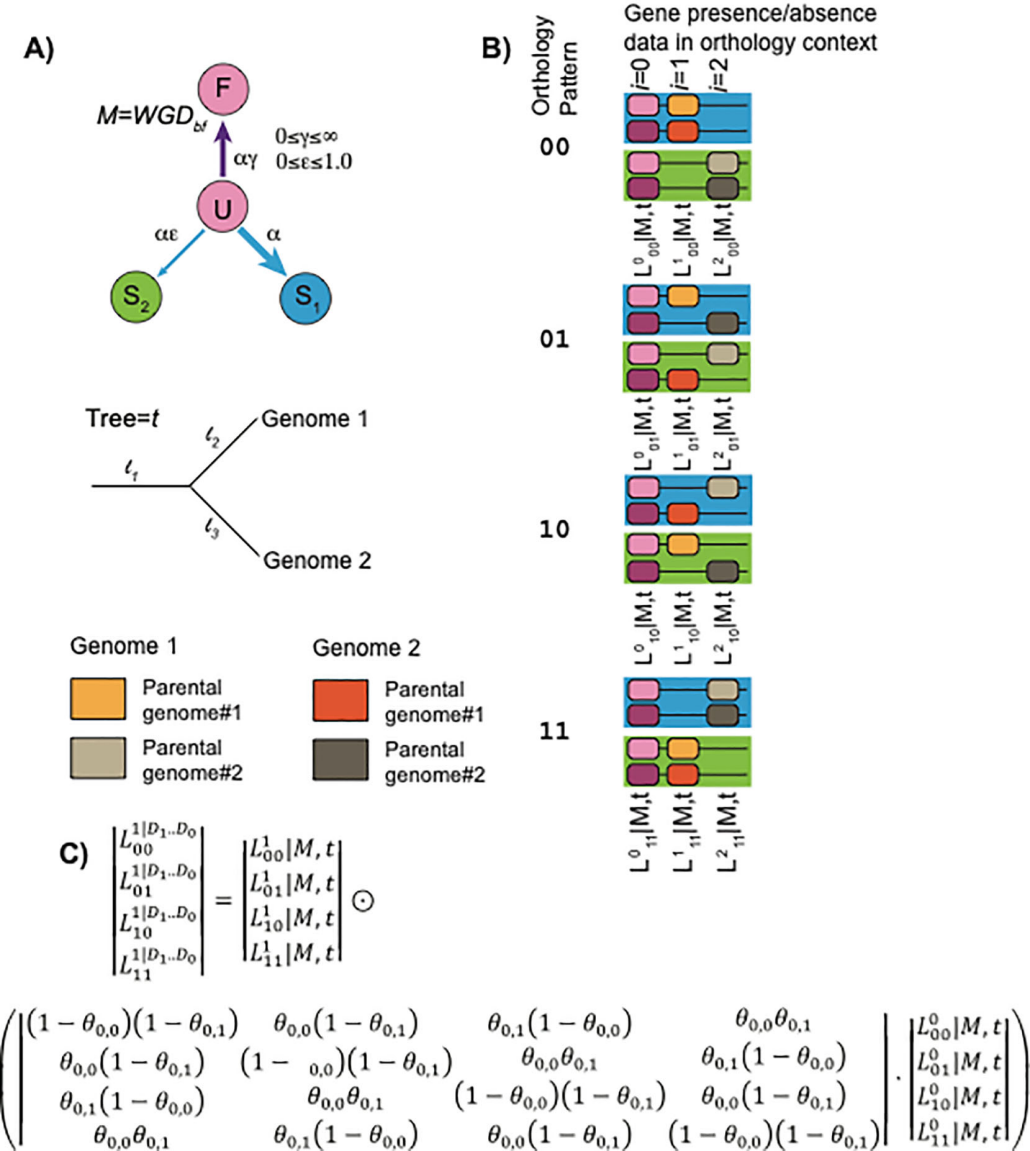
Modeling duplicate gene loss after polyploidy. **A)** Following Lewis (2001), ^120^ a discrete state model *M* allows an ancestral position to be duplicated ( **D**), single copy ( **S**_**1**_ or **S**_**2**_) or a fixed duplicate ( **D**_**f**_). Transitions between these states occur at rates proportional to model parameters ,ɛ, and γ. Losses occur along an assumed phylogenetic tree *t* with branch lengths *l* *_1_..l _t_.* The extant genomes are phased into a series of homologous columns or *pillars*: each genome may have one or two homologs present at a pillar (a state for complete homolog absence will be added to future versions of POInT). Different parental subgenomes within an extant genome can be distinguished (orange verses tan) but subgenome identities between the genomes are unknown. **B)** For *N = 2* polyploid genomes, there are *2* *^N^* possible orthology relations. At each pillar *i*, we can compute the likelihood of the observed gene presence and absence data for a given orthology pattern XX using the model *M* and the tree *t: L* *^i^_xx_* *|M,t.* **C)** Using the synteny relationships, the values *L* *^i^_00_* *|M,t* .. *L* *^i^_11_* *|M,t* can be conditioned on *L* *^i-1^_00_* *|M,t* .. *L* *^i-1^_11_* *|M,t* with a transition probability matrix Θ. The elements of Θ depend on Θ _i,g_, where *i* is the pillar number and *g* is the genome. If synteny in maintained between pillars *i* and *i+1* for genome *g,* Θ _i,g_= Θ ^M^, a global constant estimated by maximum likelihood (0≤Θ ^M^≤1). Otherwise Θ _i,g_=0.5, meaning the orthology pattern at *i* is independent of that at *i-1.* This equation can be applied recursively to compute the likelihood of the entire dataset with standard hidden Markov model approaches ^121^: the ⨀ operator represents an element-wise vector product. The tree branch lengths and model parameters are estimated from the data by maximum likelihood using standard numerical techniques. ^125^ This figure is an original figure produced by the authors for this review article.

One example of the power of combining syntenic information with models of duplicate gene gain and loss can be seen with POInT (the Polyploid Orthology Inference Tool) ^118^^,^^119^ Assuming that the duplicate products of a WGD are known through syntenic information (as in Figure 2), one can use the generic discrete character evolutionary model of Paul Lewis (2001) ^120^ to model the preservation or loss of duplicate copies in different genomes that share this WGD ( Figure 3A). Briefly, the loss model presumes all loci (or *pillars*) start in a duplicated state **D** and then can undergo fates such as loss (resulting in states **S**_**1**_ or **S**_**2**_) or becoming fixed ( **D**_**f**_). Such a model can be applied to the duplicate presence and absence data for a group of genomes sharing the polyploidy. However, the difficulty arises that the orthology assignments between those genomes is unknown. POInT hence computes the likelihood of the observed gene presence/absence data at each pillar for all possible orthology relationship under a duplicate loss model. It then uses a hidden Markov model to condition that set of likelihoods at the current pillar *i* on those from pillars 0.. *i-1-* using a transition matrix Q. ^121^ The elements of Q are determined by whether or not synteny is preserved between *i-1* and *i* in each genome. ^107^

Once such a framework is in place, standard likelihood ratio tests ^122^ can be used to test hypotheses about the evolution of polyploid genomes such as what fraction of the duplicates appear to have become fixed ^118^ or whether one of the two parental genomes from an allopolyploid is favored when duplicates are lost. ^119^ This second pattern, termed *biased fractionation*, is likely indicative of an allopolyploidy ^106^ and raises questions as to whether the subgenomes of allopolyploid hybrids are functionally compatible. ^77^

## Concluding Thoughts

A number of models have been generated that describe different levels of duplicate gene retention with different levels of mechanistic detail and as standalone models for individual problems, or as models that are integrated with other problems. Two key elements of duplicate gene retention are coding sequence function modeled using summary statistics (like dN/dS) or with Markov models describing increasing layers of complexity and expression evolution. These can also include models for syntenic position. While some of the Markov models attempt to integrate the two layers of evolution reflecting coding sequence function and expression in a sophisticated manner, other approaches either use a simpler unifying factor, like [P] in a biophysical model or treat them independently or without differential specification. Work in these directions is making substantial progress in capturing biological realism. Modeling of duplicate gene retention can converge with the broader modeling frameworks being developed for the genotype-phenotype map. While much of this modeling is in the realm of additive statistical association, the field of computational systems biology includes modeling frameworks that add another layer to the genotype-phenotype map that haven’t been touched much at the boundaries of the duplicate gene retention modeling field. Mechanistic models for gene expression evolution will also be fruitful in this field. There is a lot of room to keep expanding these modeling frameworks as genomic and other omic data accumulates for species and underlying populations and as biological domain-specific modeling improves that can improve mechanistic duplicate gene retention models.

## Data Availability

No data are associated with this article.
