# Supplementary material for: Models for the retention of duplicate genes and their biological underpinnings
Source: F1000Res. 2024 Feb 12;12:1400. Originally published 2023 Oct 24. [Version 2] doi: 10.12688/f1000research.141786.2 (PMC10762295; doi:10.12688/f1000research.141786.2)
Supplement: Peer review history

# Reviewer report: Reviewer response for version 1

**DOI:** 10.5256/f1000research.155263.r228194

**Published:** 2024-01-02

**Authors:** Arturo Becerra

**Affiliations:** 1 Universidad Nacional Autónoma de México, Mexico City, Mexico

The authors have provided a succinct yet comprehensive overview of the primary models governing the retention of duplicate genes—a critical biological process. The manuscript is well-crafted and explores a subject of significant originality, mainly as there are few or no existing text reviews of this nature. However, I have a minor concern and a suggestion:

 The text appears densely packed given the format constraints, resulting in brief coverage of specific models, such as the Dosage Imbalance Cost. While I understand the limitations imposed by the format and the challenge of expanding on each model, I propose including a table or images that summarize the characteristics of these models, both mathematically and biologically. This addition would greatly aid in providing readers with a more precise and concise understanding of the models.

 Minor point.

 Page 7, third paragraph, last line: 

 "including all vertebrates and flowering plants, as well as lineages of yeasts, ciliates and other plants."

 Change by:

 ... including all vertebrates, flowering plants, lineages of yeasts, and ciliates.

Is the review written in accessible language?

Yes

Are all factual statements correct and adequately supported by citations?

Yes

Are the conclusions drawn appropriate in the context of the current research literature?

Yes

Is the topic of the review discussed comprehensively in the context of the current literature?

Yes

Reviewer Expertise:

Early evolution of life

I confirm that I have read this submission and believe that I have an appropriate level of expertise to confirm that it is of an acceptable scientific standard.

# Reviewer report: Reviewer response for version 1

**DOI:** 10.5256/f1000research.155263.r218598

**Published:** 2023-11-22

**Authors:** Nadia El-Mabrouk

**Affiliations:** 1 Département d'informatique et de recherche opérationnelle, Université de Montréal, Montréal, Quebec, Canada

This is an interesting review on the dynamic of gene retention and loss, depending on gene structure, gene function, the mechanism creating the duplicated copies, and other biological constraints. It presents various mathematical models characterizing such gene duplicates retention/loss dynamics in a context-dependent way. In particular, the difference between gene duplicates evolution depends on whether gene copies result from small scale duplication or whole genome duplication (WGD). The last part of the paper also explains the probabilistic and algorithmic ways of predicting WGD events.

 My main criticism is that the review intermingles a variety of biological concepts and mathematical models, with no clear subdivision. The introduction should clearly explain the objective of the review and how it is organized. The content should be subdivided into coherent subsections. I would suggest adding a table with all the various constraints for gene duplicability and retention listed in the different sections, summarizing which of them are considered by which model/paper.

 In addition, it would be interesting to discuss gene retention as a function not only of small-scale duplication versus whole-genome duplication, but also as a function of the type of small-scale events: duplication of a single gene versus duplication of larger segments, tandem duplication versus retrotransposition.

 Few other comments:

- Mathematical formulae should be better written. This may be a problem linked to F1000Research's writing constraints? Writing “1-(0.5)^(f-1)” or event 1/2N is unclear. Is it 1/(2N) or (1/2)N?
- Although the target audience should be familiar with most of the biological and mathematical terms used, some are less obvious and should be explained, such as “heteromultimerization partners”.
- On page 7, correct the sentence: “The relative frequency of formation of these two types of polyploids many be approximately equal”. I guess “many” should be replaced by “may”.
- On the same page, 6th line before end: “As described, selection from various sources can also play a role.” As described where?
- First part of Figure 2: I guess the segments, for each genome, above and below that for L. oculatus correspond to the two paralogous syntenies? That should be specified.
- Caption of Figure 3: “Transitions between these states occur at rates proportional to model parameters ?, ɛ, and γ”. “?” should be corrected.

Is the review written in accessible language?

Yes

Are all factual statements correct and adequately supported by citations?

Yes

Are the conclusions drawn appropriate in the context of the current research literature?

Yes

Is the topic of the review discussed comprehensively in the context of the current literature?

Yes

Reviewer Expertise:

Computational biology, phylogeny, gene/species tree reconciliation.

I confirm that I have read this submission and believe that I have an appropriate level of expertise to confirm that it is of an acceptable scientific standard.
